# Integral terminal sliding mode fault tolerant control of quadcopter UAV systems

**DOI:** 10.1038/s41598-024-61273-2

**Published:** 2024-05-11

**Authors:** Ngoc P. Nguyen, Phongsaen Pitakwachara

**Affiliations:** Department of Mechanical Engineering, Faculty of Engineering, Chulalongkorn University, Bangkok, 10330 Thailand

**Keywords:** Sliding mode control, Actuator fault estimation, Sensor fault estimation, Fault tolerant control, Adaptive control, Nonlinear system, Mechanical engineering, Aerospace engineering

## Abstract

The article presents an active fault-tolerant control scheme with an integral terminal sliding mode controller for the UAV systems. This scheme effectively addresses saturation issues, disturbances, and sensor and actuator faults. Initially, the quadcopter UAV's model is represented in state space form. Subsequently, an augmented system incorporating auxiliary states from sensor faults is developed. An adaptive sliding mode observer is proposed for estimating the actuator and sensor faults. The integral terminal sliding mode fault-tolerant control, designed for altitude and attitude regulation, relies on fault estimation data. In contrast, a cascade proportional-integral-derivative (PID) controller is employed for position control. Simulation results demonstrate the superiority of the proposed method over existing control algorithms.

## Introduction

In recent years, unmanned aerial vehicles (UAVs) have seen a surge in usage and development, primarily due to their potential applications in surveillance, environmental monitoring, military operations, entertainment, and search and rescue missions. The quadrotor, a small rotary UAV, has particularly attracted attention in both scientific and industrial communities, thanks to its agility, compact size, simple structure, affordability, and ability for vertical takeoff and landing. The quadcopter UAV has demonstrated its versatility in various technologies, including formation flights for military purposes, fault prognosis, obstacle avoidance, object tracking, and autonomous navigation. As for UAVs, because many real applications may bring hazardous environments, the operation motor–propeller and navigation sensor systems are prone to faults, undesirable performance degradation, or even instability. When a fault occurs in the sensor system, incorrect feedback will be injected into the control system, affecting to the accuracy of control signals. The failure of an actuator deteriorates control performance and affects the stability and safety of UAVs, which may cause catastrophic accidents. Consequently, enhancing reliability and safety has become a paramount concern for UAV operations. One may address these issues by modifying the UAV propulsion system to be redundant such as employing additional sensors and actuators^1^. On the other hand, fault-tolerant control (FTC) techniques have been widely adopted to preserve an acceptable level of system performance and stability in the presence of faults. This article specifically examines sensor and actuator faults which are crucial in controlling the motion of the UAV to achieve the goal.

One way to accommodate this problem is to use the Passive Fault Tolerant Control (PFTC) technique to address faults in quadcopter UAVs without the need for fault estimation information. PFTC is a robust controller design that has been widely used for UAVs. For instance, an adaptive fuzzy system^2^ was developed as a compensator to tackle faults and nonlinearities in the system. This approach is particularly effective in overcoming instability issues arising from high adaptation rates. A feedback linearization-based fault-tolerant control method^3^ was specifically designed for quadrotors experiencing rotor failure. In^4^, an FTC design using neural networks and adaptation laws were proposed for nonlinear modeling UAV. Furthermore^5,6^, developed FTC based on an adaptive sliding mode approach. These works can maintain desired system performance in both fault-free and faulty scenarios. However, it's important to note that in PFTC methods, the magnitude of faults is limited to a certain range.

To address the limitations of PFTC methods, an Active Fault Tolerant Control (AFTC) approach has been proposed, which incorporates fault diagnosis (FD) and an FTC unit. The FD component is a crucial aspect of the AFTC system, and numerous research have applied this technique to UAV systems. In^7,8^, a fault estimation strategy based on Thau observer was designed to assess the loss of control effectiveness in the control inputs of a quadrotor. A method for estimating faults in each actuator using the Kalman filter algorithm was presented in^9^. Sliding mode observer and an adaptive law was used to estimate actuator faults^10^. Actuator fault was estimated based on $H_{\infty }$-observer in^11^ for a quadrotor helicopter, while in^12^, actuator fault diagnosis was done through neural network.

The above works predominantly examines actuator fault estimation, with less emphasis on sensor fault identification. Only a few studies have addressed fault estimation in the sensor systems of quadrotors. For instance, Avram et al.^13^ designed a FD algorithm for the inertial measurement unit (IMU) of a quadrotor. In^14^, a sensor FD method is proposed based on an index performance approach. The work in^15^ applied the Kalman filter algorithm for diagnosing sensor faults in a quadcopter. These studies demonstrate promising results in fault estimation for UAVs. Therefore, the objective of this work is to develop a controller based on the obtained fault estimation information, i.e. both the AFTC and FTC systems will be designed. The resulting system will enhance the reliability and effectiveness of the UAV by accurately identifying and compensating for both sensor and actuator faults.

Several AFTC techniques have been developed for quadrotor UAVs, with each primarily focusing on either sensor or actuator faults, but not both. For the actuator fault focus, Wang et al.^16^ used recurrent neural network and sliding mode control (SMC) to handle the actuator fault. The FTC with control allocation and sliding mode scheme was proposed in^17^ for actuator fault. The method reallocates control signals to healthy actuators based on their effectiveness levels. The AFTC scheme in^18^ combined the sliding mode observer with SMC. Actuator fault was estimated and used for system reconfiguration. The work in^19^ focused on AFTC for complete loss of actuator functionality. A sliding mode FTC with linear observer-based fault detection for severe faults was proposed^20^, while yaw control was compromised for reconfiguration during fault. $H_{\infty }$-based actuator fault estimation and fault reconfiguration were presented in^21^. An augmented FTC method was proposed for the attitude system^22^ under partial loss of effectiveness in actuators. Also, an improved integral sliding mode FTC for hypersonic vehicles under actuator fault was proposed in^23^. For the sensor fault focus,^24^ suggested sensor FTC in the presence of external disturbance. Sensor fault estimation was injected to the PID controller for fault accommodation. The work in^25^ addressed sensor fault in the attitude system using a sliding mode observer and integral SMC. A fault compensation algorithm with a feedback controller and unknown input observer was presented in^26^. Sensor fault-tolerant control using sliding mode disturbance observer was proposed in^27^.

Terminal sliding mode control (TSMC) is a recently proposed control method. The main advantage of TSMC is that it provides finite-time convergence based on the traditional design of SMC, which can readily be applied to nonlinear systems and robotic systems^28–30^. The authors in^31^ propose integral TSMC for UAV to handle disturbance, uncertainties, and actuator fault. The use of adaptive controller in this method can eliminate the upper bounded information of disturbances and uncertainties. This work considered actuator faults in the model of attitude and altitude of the UAV, but it does not address the sensor faults in controller design.

In^32,33^, TSMC approach was suggested for UAV to address external disturbances and uncertainties. However, sensor and actuator faults were not examined in this approach. While above approaches show promise in fault estimation and control reconfiguration for UAVs, they primarily focus on either sensor or actuator faults separately. In^34^, fault tolerant control of UAV is presented with considering sensor and actuator fault, but this method does not provide fault diagnosis results. Few recent studies^35–37^ consider fault tolerant control of UAV under four cases of sensor and actuator faults but these papers did not present the performance of altitude, position, and fault diagnosis results. Motivation for this paper arises from this gap, aiming to develop an AFTC method that addresses faults in both sensor and actuator systems simultaneously, enhancing the reliability and functionality of UAV systems. This article proposes an Active Fault Tolerant Control (AFTC) method for quadrotors comprising of a fault diagnosis observer and an integral terminal sliding mode control (ITSMC). Key contributions of this work are as follows:

- The proposed method can tolerate both actuator and sensor faults in quadrotor UAV, while most of current studies can deal with only actuator faults or sensor faults.
- Different to^33^, our work consider fault estimation, actuator fault, and sensor fault in the controller design as an AFTC system. Unlike^34^, this work introduces the fault estimation scheme for both sensor and actuator system. The controller uses these estimated values to provide more decisive control output for the quadrotor during the trouble.
- The combination radial basis function neural network with integral sliding mode control and adaptive law not only address sensor and actuator faults but also enhance robustness against the system uncertainties.
- Actuator saturation is considered in the controller design, which make the controller be more realistic and applicable to actual systems.
- Stability of the closed loop system is rigorously validated using the Lyapunov theorem. Since errors in the sensor and actuator systems are addressed explicitly, overall system is robust to uncertainties and disturbances.

Organization of the paper is as follow. We begin by developing the quadrotor dynamic equations. Based on the attitude and altitude dynamics, the sensor and actuator fault diagnosis system are proposed in “System modeling and fault diagnosis” section. An integral sliding mode fault-tolerant controller leveraging the estimated fault signals is presented in “Fault tolerant control design” section. The effect of the control input saturation is addressed. In “Position control design” section, a simple PID control law is used for controlling the translational movement of the quadrotor. Simulations are performed in “Simulations” section under various conditions, and comparisons with existing controller are conducted. “Conclusions” section concludes the study.

## System modeling and fault diagnosis

### Modeling quadrotor dynamics

**Figure 1:**
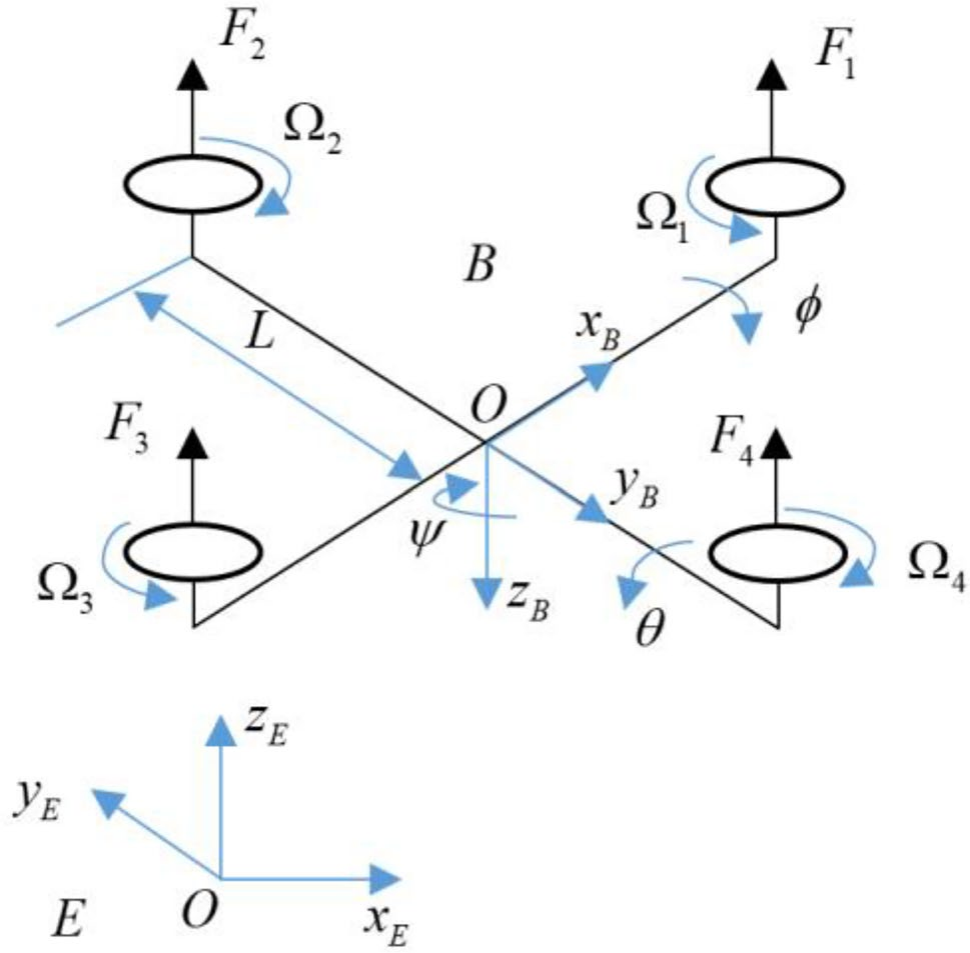
Configuration of the quadrotor.

Dynamic modeling of the quadrotor has been developed in many previous works, e.g. in^8–12^. Essential coordinate frames of quadrotor system consist of the Earth frame (E) and the body frame (B) as shown in Fig. 1. The roll, pitch, and yaw angles are respectively defined as $\phi ,\,\theta ,\,\psi \in \left( { - \pi /2,\pi /2} \right)$. Also, define $x,y,z\in {\mathbb{R}}$ as the position coordinates of the quadrotor in E. Nonlinear dynamic model of the quadcopter can be expressed as follow:

1 $$\begin{aligned} \ddot{\phi } & = \frac{{I_{2} - I_{3} }}{{I_{1} }}\dot{\theta }\dot{\psi } + \dot{\theta }\frac{{I_{\Omega } }}{{I_{1} }}\Omega_{m} + \frac{{U_{2} }}{{I_{1} }} + d_{\phi } \hfill \\ \ddot{\theta } & = \frac{{I_{3} - I_{1} }}{{I_{2} }}\dot{\phi }\dot{\psi } - \dot{\phi }\frac{{I_{\Omega } }}{{I_{2} }}\Omega_{m} + \frac{{U_{3} }}{{I_{2} }} + d_{\theta } \hfill \\ \ddot{\psi } & = \frac{{I_{1} - I_{2} }}{{I_{3} }}\dot{\phi }\dot{\theta } + \frac{{U_{4} }}{{I_{2} }} + d_{\psi } \hfill \\ \ddot{x} & = U_{1} (\cos \phi \sin \theta \cos \psi + \sin \phi \sin \psi )/m \hfill \\ \ddot{y} & = U_{1} (\cos \phi \sin \theta \sin \psi - \sin \phi \cos \psi )/m \hfill \\ \ddot{z} & = - g + \frac{{U_{1} \cos \phi \cos \theta }}{m} \hfill \\ \end{aligned}$$

where $\Omega_{m}$ and control inputs $U_{i} \,,i = 1{\text{ to }}4\,$ are defined as

2 $$\left[ \begin{aligned} \Omega_{m} \hfill \\ U_{1} \hfill \\ U_{2} \hfill \\ U_{3} \hfill \\ U_{4} \hfill \\ \end{aligned} \right] = \left[ \begin{aligned} & - \Omega_{1} + \Omega_{2} - \Omega_{3} + \Omega_{4} \hfill \\ & F_{1} + F_{2} + F_{3} + F_{4} \hfill \\ & (F_{4} - F_{2} )L \hfill \\ & (F_{1} - F_{3} )L \hfill \\ & - \tau_{1} + \tau_{2} - \tau_{3} + \tau_{4} \hfill \\ \end{aligned} \right]$$

$F_{i} = b\Omega_{i}^{2}$, $\tau_{i} = e\Omega_{i}^{2}$, $\Omega_{i}$ is the angular speed of $i^{th}$ motor, $g = 9.81$ m/s^2^, and $d_{\phi } ,\,d_{\theta } ,\,d_{\psi }$ are disturbances in roll, pitch and yaw angle respectively. Remaining parameters of the quadrotor system are shown in Table 1.

**Table 1:** Parameter of quadrotor.

System parameters	Descriptions
$I_{1} ,\,\,I_{2} ,\,\,I_{3}$ (kg/m^2^)	Inertia moments along $x,\,y,$ and $z$ directions in the earth frame
$m\,({\text{kg}})$	Total mass
$L\,({\text{m}})$	Arm length
$b\,({\text{Ns}}^{2} )$	Thrust coefficient
$e\,({\text{Nms}}^{2} )$	Drag coefficient
$I_{\Omega }$ (kg/m^2^)	Inertia moment of motor

Define

$$\begin{aligned} & X_{p} = \left[ {\begin{array}{*{20}c} z & \phi & \theta & \psi & {\dot{z}} & {\dot{\phi }} & {\dot{\theta }} & {\dot{\psi }} \\ \end{array} } \right]^{T} ,\,  \\ & u = \left[ {\begin{array}{*{20}c} {\tau_{z} } & {U_{2} } & {U_{3} } & {U_{4} } \\ \end{array} } \right]^{T} ,\,  \\ & d = \left[ {\begin{array}{*{20}c} 0 & {d_{\phi } } & {d_{\theta } } & {d_{\psi } } \\ \end{array} } \right]^{T} ,\, \\ & \tau_{z} = U_{1} /m - g. \end{aligned}$$

Then, the state equations of the attitude and altitude for the quadrotor may be expressed as

3 $$\left\{ \begin{aligned} \dot{X}_{p} (t) & = A_{p} X_{p} (t) + B_{p} u(t) + g(X_{p} ,t) + D_{p} d(t) \hfill \\ Y_{p} (t) & = C_{p} X_{p} (t) \hfill \\ \end{aligned} \right.$$

where

$$\begin{aligned} A_{p} & = \left[ {\begin{array}{*{20}c} 0 & {I_{4} } \\ 0 & 0 \\ \end{array} } \right],\,B_{p} = \left[ {\begin{array}{*{20}c} {0_{4 \times 4} } & {diag(1,I_{1}^{ - 1} ,I_{2}^{ - 1} ,I_{3}^{ - 1} )} \\ \end{array} } \right],\,C_{p} = I_{8} ,\,  \\ D_{p} & = \left[ {\begin{array}{*{20}c} {0_{5 \times 4} } & {I_{3 \times 4} } \\ \end{array} } \right],\,c_{1} = \frac{{I_{2} - I_{3} }}{{I_{1} }},c_{2} = \frac{{I_{3} - I_{1} }}{{I_{2} }},\,c_{3} = \frac{{I_{1} - I_{2} }}{{I_{3} }},\,  \\ c_{4} & = \frac{{I_{\Omega } }}{{I_{1} }},\,\,c_{5} = \frac{{I_{\Omega } }}{{I_{2} }},\,\,b_{z} = \cos \phi \cos \theta   \\ g(X_{p} ,t) = \left[ {\begin{array}{l} {0_{4 \times 1} } \\ {(b_{z} - 1)U_{1} /m} \\ {c_{1} \dot{\theta }\dot{\psi } + c_{4} \dot{\theta }\Omega_{m} } \\ {c_{2} \dot{\phi }\dot{\psi } - c_{5} \dot{\phi }\Omega_{m} } \\ {c_{3} \dot{\phi }\dot{\theta }} \\ \end{array} } \right]. \end{aligned}$$

Equation (3) will be used as a basis in designing the sensor and actuator fault diagnosis system.

### Fault diagnosis system design

In this section, the observer-based sensor and actuator fault diagnosis system are proposed. Since the faults occur in the attitude system due to poor actuators and noisy IMU sensor, the sensor fault diagnosis will focus solely the attitude system. From (3), state space model of the attitude and altitude system under the presence of angular rate sensor and actuator faults may be expressed as

4 $$\begin{aligned} \dot{X}_{p} & = A_{p} X_{p} + B_{p} (u + u_{a} ) + g(X_{p} ,t) + D_{p} d \hfill \\ Y_{p} & = C_{p} X_{p} + T_{p} f_{s} \hfill \\ \end{aligned}$$

where $u_{a}$ and $f_{s}$ represent the actuator and sensor fault vector, $T_{p}$ is sensor fault matrix with appropriate dimension.

Later, the estimation of $u_{a}$ and $f_{s}$ will be employed by the fault tolerant controller to accommodate for the occurring fault. The following assumptions and lemma are necessary for deriving the fault diagnosis system.

#### Assumption 1

^10^ The continuous nonlinear system $g(x,t)$ is assumed to be Lipschitz, that is $\left\| {g(x_{1} ,t) - g(x_{2} ,t)} \right\| \le \gamma \left\| {x_{1} - x_{2} } \right\|$, where $\gamma$ is the known positive constant.

#### Assumption 2

The external disturbance $d(t)$ is norm bounded, $i.e.$ $\left\| {d(t)} \right\| < M$.

#### Assumption 3

The pair $(A_{p} ,B_{p} )$ is controllable and $(A_{p} ,C_{p} )$ is detectable.

#### Lemma 1

^21^ For given a positive scalar $\varepsilon$ and a positive definite matrix $P$, the following inequality holds.

5 $$2{x}^{T}y\le \frac{1}{\varepsilon }{x}^{T}Px+\varepsilon {y}^{T}{P}^{-1}y x,y\in {\mathbb{R}}^{n}$$

#### Lemma 2

^33^ For a continuous positive-definite Lyapunov function $V(t)$, if it satisfies $\dot{V}(t) \le - cV^{d} - mV$ with $c > 0,\,m > 0,\,0 < d < 1$, then $V(t)$ converges to zero in finite time $T_{f}$ with

$$T_{f} = \frac{1}{m(1 + d)}\ln \left( {\frac{{mV^{1 - d} (0) + c}}{c}} \right)$$

The system (4) can be reformulated as the augmented system:

6 $$\begin{aligned} \dot{X} & = AX + B(u + u_{a} ) + g(X,t) + Dd + N\dot{f}_{s} \hfill \\ Y & = CX \hfill \\ \end{aligned}$$

where $$X = \left[ {\begin{array}{*{20}c} {X_{p}^{T} } & {f_{s}^{T} } \\ \end{array} } \right]^{T}$$, $$A = \left[ {\begin{array}{*{20}c} {A_{p} } & 0 \\ 0 & 0 \\ \end{array} } \right]$$, $$B = \left[ {\begin{array}{*{20}c} {B_{p} } \\ 0 \\ \end{array} } \right]$$, $$C = \left[ {\begin{array}{*{20}c} {C_{p} } & {T_{p} } \\ \end{array} } \right]$$, $$D = \left[ {\begin{array}{*{20}c} {D_{p} } \\ 0 \\ \end{array} } \right]$$, $$g(X,t) = \left[ {\begin{array}{*{20}c} {g(X_{p} ,t)} \\ 0 \\ \end{array} } \right]$$, $$N = \left[ {\begin{array}{*{20}c} 0 \\ I \\ \end{array} } \right]$$.

Let us define

7 $$\begin{aligned} e_{X} & = \hat{X} - X,\, \hfill \\ e_{Y} & = \hat{Y} - Y,\, \hfill \\ e_{u} & = \hat{u}_{a} - u_{a} , \hfill \\ \Delta g & = g(\hat{X},t) - g(X,t) \hfill \\ \end{aligned}$$

Accordingly, the following adaptive fault diagnosis observer^21^ is proposed to estimate the system (6)

8 $$\begin{aligned} \dot{\hat{X}} & = A\hat{X} + Bu + g(\hat{X},t) + Dv + B\hat{u}_{a} - L_{0} (\hat{Y} - Y) \hfill \\ \hat{Y} & = C\hat{X} \hfill \\ \end{aligned}$$

where $\hat{X}$ is denoted as state observer vector, $\hat{Y}$ is the output vector, and $L_{0}$ is the observer gain. Vector $v$ is defined as:

9 $$v = - \kappa \frac{{K_{2} e_{Y} }}{{\left\| {K_{2} e_{Y} } \right\| + \delta }}$$

where $\delta$ is a small positive constant, $\kappa$ is a positive gain such that $\kappa > M$. Corresponding observer error dynamics may be expressed as:

10 $$\begin{aligned} \dot{e}_{X} & = (A - L_{0} C)e_{X} + \Delta g + D(v - d) + Be_{u} - N\dot{f}_{s} \hfill \\ e_{Y} & = Ce_{X} \hfill \\ \end{aligned}$$

#### Theorem 1

If there exist symmetric positive matrices $P,\,\,G$ and matrices $Y,\,K_{1} ,\,K_{2}$ such that the following conditions (11–13) and adaptive law (14) hold

11 $$\Phi = \left[ {\begin{array}{*{20}c} \Pi & 0 & P & {PN} \\ 0 & G & 0 & 0 \\ P & 0 & { - \gamma^{ - 2} I} & 0 \\ {(PN)^{T} } & 0 & 0 & { - \varepsilon^{ - 1} I} \\ \end{array} } \right] < 0$$

12 $$B^{T} P = K_{1} C$$

13 $$D^{T} P = K_{2} C$$

14 $$\dot{\hat{u}}_{a} = - \Gamma K_{1} e_{Y} (t)$$

where $\Pi = PA + A^{T} P - YC - C^{T} Y^{T} + I,\,\,L_{0} = P^{ - 1} Y$, $\gamma$ and $\varepsilon$ are positive constants. Then, the observer (8) and (14) can asymptotically estimate the states, sensor, and actuator faults.

#### Proof

Consider the Lyapunov function:

15 $$V = e_{X}^{T} Pe_{X} + e_{u}^{T} \Gamma^{ - 1} e_{u}$$

Using (10), the time derivative of $V$ may be derived as

16 $$\begin{aligned} \dot{V} & = \dot{e}_{X}^{T} Pe_{X} + e_{X}^{T} P\dot{e}_{X} + 2e_{u}^{T} \Gamma^{ - 1} \dot{e}_{u} \hfill \\ & = e_{X}^{T} \left[ {(A - L_{0} C)^{T} P + P(A - L_{0} C)} \right]e_{X} \hfill \\ & \quad + 2e_{X}^{T} P\Delta g\, + 2e_{X}^{T} PD(v - d) \hfill \\ & \quad + 2e_{X}^{T} PBe_{u} \, - 2e_{X}^{T} PN\dot{f}_{s} + 2e_{u}^{T} \Gamma^{ - 1} \dot{\hat{u}}_{a} - 2e_{u}^{T} \Gamma^{ - 1} \dot{u}_{a} \hfill \\ \end{aligned}$$

From Assumption 2, we have

17 $$\begin{gathered} 2e_{X}^{T} PBe_{u} \, + 2e_{u}^{T} \Gamma^{ - 1} \dot{\hat{u}}_{a} = 2e_{X}^{T} PBe_{u} - 2e_{u}^{T} \Gamma^{ - 1} \Gamma K_{1} e_{Y} = 0 \hfill \\ \end{gathered}$$

18 $$\begin{aligned} 2e_{X}^{T} PD(v - d) & = 2e_{X}^{T} PD\left( { - \kappa \frac{{K_{2} e_{Y} }}{{\left\| {K_{2} e_{Y} } \right\|}} - d} \right) \hfill \\ & = 2(K_{2} e_{Y} )^{T} \left( { - \kappa \frac{{K_{2} e_{Y} }}{{\left\| {K_{2} e_{Y} } \right\|}} - d} \right) \hfill \\ & < 2\left\| {K_{2} e_{Y} } \right\|( - \kappa + M) < 0 \hfill \\ \end{aligned}$$

Furthermore, using Assumption 1 and Lemma 1, one can obtain:

19 $$2e_{X}^{T} P\Delta g\, < \gamma^{2} e_{X}^{T} PPe_{X} + e_{X}^{T} e_{X}$$

20 $$- 2e_{X}^{T} PN\dot{f}_{s} < \varepsilon e_{X}^{T} PNN^{T} P^{T} e_{X} + \varepsilon^{ - 1} f_{0}^{2}$$

21 $$\begin{aligned} - 2e_{u}^{T} \Gamma^{ - 1} \dot{u}_{a} & \le e_{u}^{T} Ge_{u} + \dot{u}_{a}^{T} \Gamma^{ - 1} G\Gamma^{ - 1} \dot{u}_{a} \hfill \\ & \le e_{u}^{T} Ge_{u} + f_{1}^{2} \lambda_{\max } (\Gamma^{ - 1} G\Gamma^{ - 1} ) \hfill \\ \end{aligned}$$

in which $f_{0}$ and $f_{1}$ are the upper bound of $\dot{f}_{s}$ and $\dot{u}_{a}$, and $\lambda_{{{\text{max}}}} \left( \cdot \right), \, \lambda_{{{\text{min}}}} \left( \cdot \right)$ denotes the max/min eigenvalue of the matrix. Substituting (17)–(21) into (16), we have

22 $$\begin{aligned} \dot{V} & \le e_{X}^{T} \left[ {(A - L_{0} C)^{T} P + P(A - L_{0} C) + \gamma^{2} PP + I} \right]e_{X} \hfill \\ & \quad + \varepsilon e_{X}^{T} PNN^{T} P^{T} e_{X} + \varepsilon^{ - 1} f_{0}^{2} \hfill \\ & \quad + e_{u}^{T} Ge_{u} + f_{1}^{2} \lambda_{\max } (\Gamma^{ - 1} G\Gamma^{ - 1} ) \hfill \\ & \le e_{X}^{T} \left[ \begin{gathered} (A - L_{0} C)^{T} P + P(A - L_{0} C) \hfill \\ + \gamma^{2} PP + I + \varepsilon PNN^{T} P^{T} \hfill \\ \end{gathered} \right]e_{X} \hfill \\ & \quad + e_{u}^{T} Ge_{u} + \beta \hfill \\ \end{aligned}$$

where $$\beta = \varepsilon^{ - 1} f_{0}^{2} + f_{1}^{2} \lambda_{\max } (\Gamma^{ - 1} G\Gamma^{ - 1} )$$.

Let $$\xi (t) = \left[ {\begin{array}{ll} {e_{X}^{T} } & {e_{u}^{T} } \\ \end{array} } \right]^{{^{T} }}$$, then $\dot{V} \le \xi^{T} \Phi \xi + \beta$ in which $$\Theta = \left[ {\begin{array}{*{20}c} {(A - LC)^{T} P + P(A - LC) + \gamma^{2} PP + I + \varepsilon PNN^{T} P^{T} } & 0 \\ 0 & G \\ \end{array} } \right]$$.

When $\Theta < 0$, then $\dot{V} < - \sigma \left\| \xi \right\|^{2} + \beta$, where $\sigma = \lambda_{\min } ( - \Theta )$, which means that $(e_{X} ,e_{u} )$ asymptotically converges to a small set around 0 according to Lyapunov stability theory. Therefore, estimation errors of the fault and the state are uniformly bounded. This proves the stability of the observer error dynamics.

It should be noted that $\Theta$ is a standard linear matrix inequalities (LMI) form. By applying Schur complement lemma for LMI^38,39^, we can achieve the form in (11).

## Fault tolerant control design

With the estimated sensor and actuator fault signals, we propose an integral terminal sliding mode fault-tolerant controller for controlling the attitude and altitude of the quadrotor. The nonlinear model of the attitude and altitude system under input saturation can be expressed as:

23 $$\left\{ \begin{aligned} \dot{\overline{x}}_{1} & = \overline{x}_{2} + f_{s} \hfill \\ \dot{\overline{x}}_{2} & = \overline{g} + \overline{b}sat(u) + \overline{b}sat(u_{a} ) + \overline{d} \hfill \\ \overline{y} & = \overline{x}_{1} \hfill \\ \end{aligned} \right.$$

where $$\overline{x}_{1} = \left[ \begin{gathered} x_{11} \hfill \\ x_{12} \hfill \\ x_{13} \hfill \\ x_{14} \hfill \\ \end{gathered} \right] = \left[ {\begin{array}{*{20}c} z \\ \phi \\ \theta \\ \psi \\ \end{array} } \right]$$, $$\overline{x}_{2} = \left[ {\begin{array}{*{20}c} {x_{21} } \\ {x_{22} } \\ {x_{23} } \\ {x_{24} } \\ \end{array} } \right] = \left[ {\begin{array}{*{20}c} {\dot{z}} \\ {\dot{\phi }} \\ {\dot{\theta }} \\ {\dot{\psi }} \\ \end{array} } \right]$$, $$\overline{g} = \left[ {\begin{array}{*{20}c} {g_{1} } \\ {g_{2} } \\ {g_{3} } \\ {g_{4} } \\ \end{array} } \right] = \left[ {\begin{array}{*{20}c} {\frac{1}{m}(b_{z} - 1)U_{1} } \\ {c_{1} x_{23} x_{24} + c_{4} x_{23} \Omega_{m} } \\ {c_{2} x_{22} x_{24} - c_{5} x_{22} \Omega_{m} } \\ {c_{3} x_{22} x_{23} } \\ \end{array} } \right]$$, $$\overline{d} = \left[ {\begin{array}{*{20}c} {d_{1} } \\ {d_{2} } \\ {d_{3} } \\ {d_{4} } \\ \end{array} } \right] = \left[ {\begin{array}{*{20}c} 0 \\ {d_{\phi } } \\ {d_{\theta } } \\ {d_{\psi } } \\ \end{array} } \right]$$, $$f_{s} = \left[ {\begin{array}{*{20}c} {f_{{s_{1} }} } \\ {f_{{s_{2} }} } \\ {f_{{s_{3} }} } \\ {f_{{s_{4} }} } \\ \end{array} } \right]$$, $$\overline{b} = diag\left[ {\begin{array}{*{20}c} {b_{1} } \\ {b_{2} } \\ {b_{3} } \\ {b_{4} } \\ \end{array} } \right] = diag\left[ {\begin{array}{*{20}c} 1 \\ {1/I_{1} } \\ {1/I_{2} } \\ {1/I_{3} } \\ \end{array} } \right]$$, $$u = \left[ {\begin{array}{*{20}c} {u_{1} } \\ {u_{2} } \\ {u_{3} } \\ {u_{4} } \\ \end{array} } \right] = \left[ {\begin{array}{*{20}c} {\tau_{z} } \\ {U_{2} } \\ {U_{3} } \\ {U_{4} } \\ \end{array} } \right]$$, $$u_{a} = \left[ {\begin{array}{*{20}c} {u_{a1} } \\ {u_{a2} } \\ {u_{a3} } \\ {u_{a4} } \\ \end{array} } \right]$$**.**

$f_{s}$ and $u_{a}$ are sensor and actuator fault determined from “Fault diagnosis system design” section.

Define $\Delta {u}_{i}=sat({u}_{i})-{u}_{i}$ and $\Delta {u}_{ai}=sat({u}_{ai})-{u}_{ai}$, then the system (23) can be rewritten as:

24 $$\left\{ \begin{aligned} & \dot{\overline{x}}_{1} = \overline{x}_{2} + f_{s} \hfill \\ & \dot{\overline{x}}_{2} = \overline{g} + \overline{b}u + \overline{b}u_{a} + \overline{\upsilon } \hfill \\ & \overline{y} = \overline{x}_{1} \hfill \\ \end{aligned} \right.$$

where $$\bar{\upsilon}=\left[\begin{array}{c}{\upsilon }_{1}\\ {\upsilon }_{2}\\ {\upsilon }_{3}\\ {\upsilon }_{4}\end{array}\right]=\left[\begin{array}{c}{\bar{b}}_{1}\Delta {u}_{1}+{\bar{b}}_{1}\Delta {u}_{a1}+{\bar{d}}_{1}\\ {\bar{b}}_{2}\Delta {u}_{2}+{\bar{b}}_{2}\Delta {u}_{a2}+{\bar{d}}_{2}\\ {\bar{b}}_{3}\Delta {u}_{3}+{\bar{b}}_{3}\Delta {u}_{a3}+{\bar{d}}_{3}\\ {\bar{b}}_{4}\Delta {u}_{4}+{\bar{b}}_{4}\Delta {u}_{a4}+{\bar{d}}_{4}\end{array}\right]$$.

The tracking error can be defined as:

25 $$\overline{e} = \overline{x}_{d} - \overline{x}_{1} = \left[ \begin{gathered} e_{1} \hfill \\ e_{2} \hfill \\ e_{3} \hfill \\ e_{4} \hfill \\ \end{gathered} \right] = \left[ \begin{gathered} z_{d} - z \hfill \\ \phi_{d} - \phi \hfill \\ \theta_{d} - \theta \hfill \\ \psi_{d} - \psi \hfill \\ \end{gathered} \right]$$

where $$\overline{x}_{d} = \left[ {\begin{array}{*{20}c} {x_{d1} } & {x_{d2} } & {x_{d3} } & {x_{d4} } \\ \end{array} } \right]^{T} = \left[ {\begin{array}{*{20}c} {z_{d} } & {\phi_{d} } & {\theta_{d} } & {\psi_{d} } \\ \end{array} } \right]^{T}$$ is the desired vector of $\overline{x}_{1}$.

Because $\dot{\overline{x}}_{1}$ from the sensor contains $f_{s}$, time derivative of the tracking error for the sliding surface must be corrected by subtracting off with the estimate of $f_{s}$, or $\hat{f}_{s}$, as

26 $$\dot{\overline{e}} = \dot{\overline{x}}_{d} - \dot{\overline{x}}_{c}$$

where $$\dot{\overline{x}}_{c} = \left[ {\begin{array}{*{20}c} {x_{c1} } & {x_{c2} } & {x_{c3} } & {x_{c4} } \\ \end{array} } \right]^{T} = \overline{x}_{2} + f_{s} - \hat{f}_{s}$$.

Sliding surface for the fault-tolerant control system is defined element-wise for $i = 1, \ldots ,4$ as:

27 $${s}_{i}={\dot{e}}_{i}+{k}_{1i}{e}_{i}+{k}_{2i}{\int }_{0}^{t}{e}_{i}^{[{q}_{i}/{p}_{i}]}d\tau$$

where $k_{1i}$, $k_{2i}$ are positive gains; $q_{i} < p_{i}$ with $p_{i}$ and $q_{i}$ are odd positive values; $e_{i}^{{[q_{i} /p_{i} ]}}$ is a function of time and defined as $e_{i}^{{[q_{i} /p_{i} ]}} = \left| {e_{i} } \right|^{{q_{i} /p_{i} }} {\text{sgn}} (e_{i} )$.

From (26) and (27), we have:

28 $$\begin{aligned} {s}_{i}& =\left({\dot{x}}_{di}-\left({x}_{2i}-{\widetilde{f}}_{si}\right)\right)+{k}_{1i}\left({x}_{di}-{x}_{1i}\right)+{k}_{2i}{\int }_{0}^{t}{e}_{i}^{\left[\frac{{q}_{i}}{{p}_{i}}\right]}d\tau \\ &=({\dot{x}}_{di}-{x}_{2i})+{k}_{1i}({x}_{di}-{x}_{1i})+{k}_{2i}{\int }_{0}^{t}{e}_{i}^{[{q}_{i}/{p}_{i}]}d\tau +{\widetilde{f}}_{si} \end{aligned}$$

where $\tilde{f}_{si} = \hat{f}_{si} - f_{si}$. The derivative of sliding surface becomes:

29 $$\begin{aligned} \dot{s}_{i} & = (\ddot{x}_{di} - \dot{x}_{2i} ) + k_{1i} (\dot{x}_{di} - \dot{x}_{ci} ) + k_{2i} e_{i}^{{[q_{i} /p_{i} ]}} + \dot{\tilde{f}}_{si} \hfill \\ & = (\ddot{x}_{di} - \dot{x}_{2i} ) + k_{1i} (\dot{x}_{di} - (x_{2i} + f_{si} - \hat{f}_{si} )) \hfill \\ & \quad + k_{2i} e_{i}^{{[q_{i} /p_{i} ]}} + \dot{\tilde{f}}_{si} \hfill \\ & = (\ddot{x}_{di} - \dot{x}_{2i} ) + k_{1i} (\dot{x}_{di} - \dot{x}_{1i} ) \hfill \\ & \quad + k_{1i} \hat{f}_{si} + k_{2i} e_{i}^{{[q_{i} /p_{i} ]}} + \dot{\tilde{f}}_{si} \hfill \\ & = (\ddot{x}_{di} - g_{i} - b_{i} u_{i} - b_{i} u_{ai} - \upsilon_{i} ) + k_{1i} (\dot{x}_{di} - \dot{x}_{1i} ) \hfill \\ & \quad + k_{1i} \hat{f}_{si} + k_{2i} e_{i}^{{[q_{i} /p_{i} ]}} + \dot{\tilde{f}}_{si} \hfill \\ \end{aligned}$$

Typically, the uncertainty term $g_{i}$ is difficult to achieve in experiment. However, it can be approximated using radial basis function neural network (RBFNN)^39^ as below:

30 $$g_{i} = W_{i}^{T} X_{i} + \delta_{i}$$

where $W_{i} \in {\mathbb{R}}^{n}$ is the optimal weight matrix, $X_{i} \in {\mathbb{R}}^{n}$ is the nonlinear function of hidden nodes, $n = 5$, $\delta_{i}$ is the approximation error. The Gaussian function is used for nonlinear function $X_{i}$ as follows:

31 $$X_{ij} (\mu ) = \exp \left( { - \frac{{\left\| {\mu - r_{j} } \right\|^{2} }}{{o_{j}^{2} }}} \right),\,\,j = 1,2,..,n$$

where $o_{j}^{2} = 7.5$ is the width of Gaussian function, $r_{j} \in {\mathbb{R}}^{2}$ is the center of Gaussian function which is chosen between -1 and 1, $$\mu = \left[ {\begin{array}{*{20}c} {\overline{e}_{i} } & {\dot{\overline{e}}_{i} } \\ \end{array} } \right]^{T}$$.

### Theorem 2

If the sliding surface is defined as (27) and the fault tolerant control law using sensor reading $\dot{\overline{x}}_{1}$ is designed as:

32 $$u_{i} = b_{i}^{ - 1} \left( \begin{aligned} & \ddot{x}_{di} - \hat{W}_{i} X_{i} - b_{i} \hat{u}_{ai} + k_{1i} (\dot{x}_{di} - \dot{x}_{1i} ) \hfill \\ & + k_{1i} \hat{f}_{si} + k_{2i} e_{i}^{{[q_{i} /p_{i} ]}} + \lambda_{i} s_{i} \hfill \\ & + h_{1i} \left| {s_{i} } \right|^{{\alpha_{i} }} sign(s_{i} ) + h_{2i} \left| {s_{i} } \right|^{{\beta_{i} }} sign(s_{i} ) \hfill \\ \end{aligned} \right)$$

and updated by:

33 $$\dot{\hat{W}}_{i} = - \gamma_{i} s_{i} X_{i}$$

where $h_{1i} ,\,h_{2i} ,\,\gamma_{i}$ are the positive gains, $\alpha_{i} > 1,\,0 < \beta_{i} < 1$. Then the system (24) converges to origin in finite time.

### Proof

Choose the Lyapunov function as

34 $$V_{i} = \frac{1}{2}s_{i}^{2} + \frac{1}{{2\gamma_{i} }}\tilde{W}_{i}^{T} \tilde{W}_{i}$$

where $\tilde{W}_{i} = W_{i} - \hat{W}_{i}$, $\hat{W}_{i}$ is the estimate of $W_{i}$.

From (28)–(32), the derivative of Lyapunov function is

35 $$\begin{aligned} \dot{V}_{i} & = s_{i} \dot{s}_{i} - \frac{1}{{2\gamma_{i} }}\tilde{W}_{i}^{T} \dot{\hat{W}}_{i} \hfill \\ &= s_{i} \left( \begin{aligned}& \delta_{i} - \tilde{W}_{i}^{T} X_{i} + b_{i} e_{ui} + \dot{\tilde{f}}_{si} - \upsilon_{i} \hfill \\ & - h_{1i} \left| {s_{i} } \right|^{{\alpha_{i} }} sign(s_{i} ) - h_{2i} \left| {s_{i} } \right|^{{\beta_{i} }} sign(s_{i} ) - \lambda_{i} s_{i} \hfill \\ \end{aligned} \right) \hfill \\ & \quad + \tilde{W}_{i}^{T} s_{i} X_{i} \hfill \\ & \le (\eta_{i} - h_{1i} \left| {s_{i} } \right|^{{\alpha_{i} }} )\left| {s_{i} } \right| - \lambda_{i} s_{i}^{2} - h_{2i} \left| {s_{i} } \right|^{{\beta_{i} + 1}} \hfill \\ \end{aligned}$$

where $$e_{ui} = \hat{u}_{ai} - u_{ai}$$, $$\eta_{i} = \left| {\delta_{i} } \right| + \left| {b_{i} e_{ui} } \right| + \left| {\dot{\tilde{f}}_{si} } \right| + \left| {\upsilon_{i} } \right|$$.

If we set $h_{1i} \left| {s_{i} } \right|^{{\alpha_{i} }} \ge \eta_{i}$ then we have:

36 $$\dot{V}_{i} \le - \lambda_{i} s_{i}^{2} - h_{2i} \left| {s_{i} } \right|^{{\beta_{i} + 1}}$$

According to (31), one obtain:

37 $$\dot{V}_{i} \le - 2\lambda_{i} V_{i} - 2^{{\frac{{\beta_{i} + 1}}{2}}} h_{2i} V_{i}^{{\frac{{\beta_{i} + 1}}{2}}}$$

Recalling Lemma 2, the terminal sliding mode surfaces (16)–(21) converge to the origin in finite time.

### Remark

The double reaching law in (32), $h_{1i} \left| {s_{i} } \right|^{{\alpha_{i} }} sign(s_{i} ) + h_{2i} \left| {s_{i} } \right|^{{\beta_{i} }} sign(s_{i} )$, provides faster convergence with reduction of chattering effect^40,41^.

## Position control design

In real applications, the position controller is designed with lower frequency compared to attitude and altitude controller because it is used to transform to desired roll and pitch angles. For simplicity, a cascade PID control law^42,43^ is used to design the translational movements of quadrotor as Eq. (38) follows:

38 $$\begin{aligned} \Delta_{x} &= k_{Px}^{out} (x_{d} - x) - \dot{x} \hfill \\ \ddot{x} & = k_{Px}^{in} \Delta_{x} + k_{Ix}^{in} \int {\Delta_{x} dt + } k_{Dx}^{in} \dot{\Delta }_{x} \hfill \\ \Delta_{y} & = k_{Py}^{out} (y_{d} - y) - \dot{y} \hfill \\ \ddot{y} & = k_{Py}^{in} \Delta_{y} + k_{Iy}^{in} \int {\Delta_{y} dt + } k_{Dy}^{in} \dot{\Delta }_{y} \hfill \\ \end{aligned}$$

where $x_{d}$ and $y_{d}$ are desired positions; $x$ and $y$ are current positions; $k_{Px}^{out}$, $k_{Py}^{out}$ are the gains of outer loop, while $k_{Px}^{in}$, $k_{Py}^{in}$, $k_{Ix}^{in}$, $k_{Iy}^{in}$, $k_{Dx}^{in}$, $k_{Dy}^{in}$ are the gains of inner loop. In addition, from (1) and the IMU readouts, we can determine the desired roll and pitch angles as:

39 $$\begin{aligned} \phi_{d} & = \sin^{ - 1} \left( {m\left( {\ddot{x}\sin \psi - \ddot{y}\cos \psi } \right)/U_{1} } \right) \hfill \\ \theta_{d} & = \sin^{ - 1} \left( {m(\ddot{x}\cos \psi + \ddot{y}\sin \psi )/U_{1} \cos \phi_{d} } \right) \hfill \\ \end{aligned}$$

**Figure 2:**
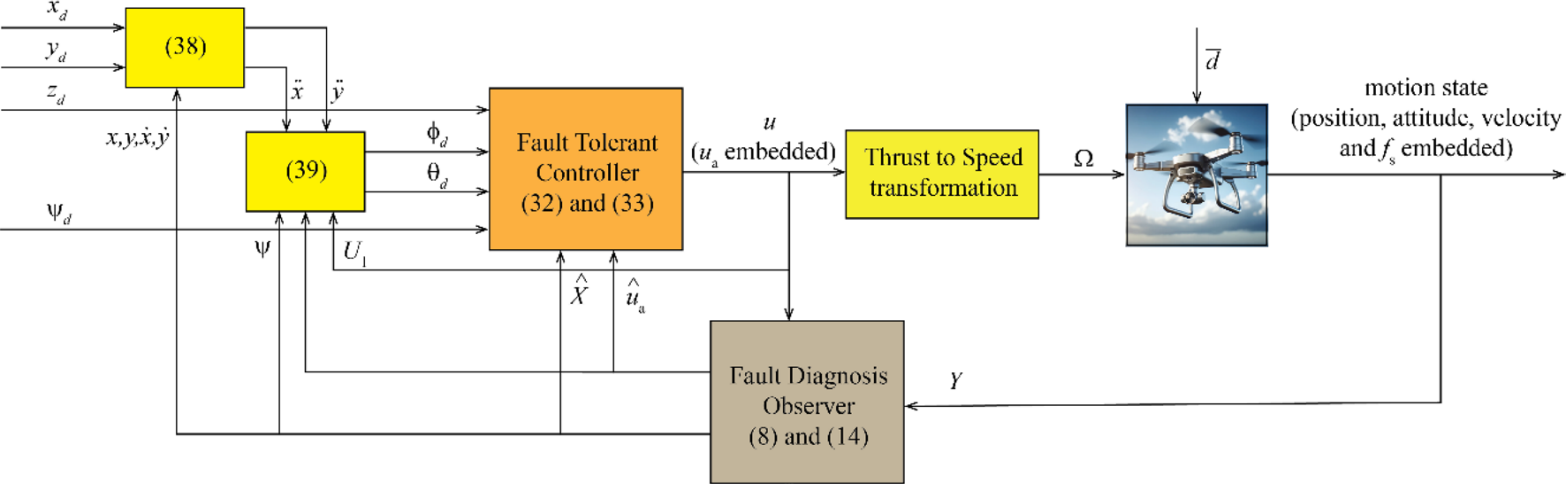
Overall block diagram.

**Figure 3:**
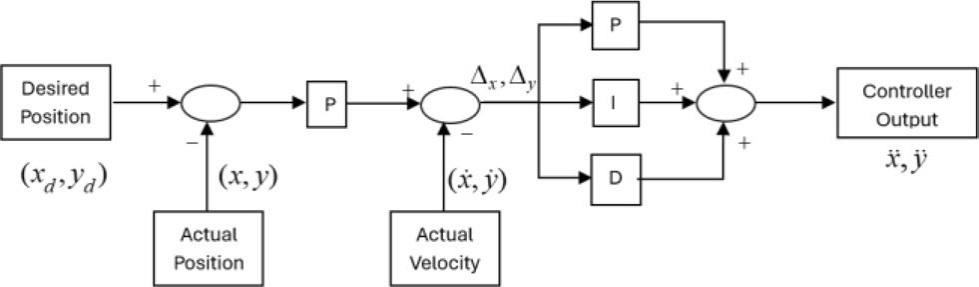
Cascade PID for position controller.

Figure 2 displays the overall block diagram of the control system, while Fig. 3 shows the cascade PID control law of Eq. 38. The attitude and altitude motion are controlled by the proposed fault tolerant observer and controller. The translational motion is controlled indirectly through the attitude controller by generating of the desired roll and pitch angles.

## Simulations

Performance of the proposed fault diagnosis observer and fault-tolerant controller is validated through a series of numerical simulations on a quadrotor system. These simulations are crucial for demonstrating the effectiveness of the new approach under various conditions and scenarios. To provide a clear comparison and highlight the strengths of the proposed controller, the ITSMC method in^33^ is used as a benchmark. The parameters of quadrotor can be summarized in Table 2.

**Table 2:** Parameter of quadrotor for simulation.

Parameters	Value	Unit
$(I_{1} ,\,\,I_{2} ,\,\,I_{3} )$	(0.004,0.004,0.0084)	$\,{\text{kgm}}^{2}$
$m$	0.74	kg
$L$	0.1	m
$b$	$2.9842 \times 10^{ - 3}$	Ns^2^
$e$	$3.2320 \times 10^{ - 2}$	Nms^2^

The sampling time of simulation is chosen as $T_{s} = 0.0025s$, which depends on the open-source flight control software of UAV^44^. The following parameters are chosen for fault diagnosis observer and fault-tolerant controller.

$$\begin{aligned} G & = 10^{ - 3} I_{4} ,\,T_{p} = 150 \times \left[ {\begin{array}{*{20}c} {0_{4 \times 4} } & {I_{4} } \\ \end{array} } \right],\,d_{\phi } = d_{\theta } = d_{\psi } = 0.2,\, \hfill \\ \Gamma & = diag(1,0.0017,0.0017,0.0017),\,\kappa = {10}^{ - 7} ,\,\varepsilon = 0.001,\,\gamma = 2 \hfill \\ \alpha_{i} & = 1.2,\,\beta_{i} = 3/5,\,q_{i} = 3,\,p_{i} = 15,\,k_{1i} = 25,\,k_{2i} = 0.1,\,h_{1i} = 1,\, \hfill \\ h_{2i} & = 10,\,k_{px} = k_{py} = 2.5,\,k_{dx} = k_{dy} = 7. \hfill \\ \end{aligned}$$

The values of $$K_{1}$$, $$K_{2}$$, $$L_{o}$$ are shown the Appendix section. The control input is saturated as: $$0 \le U_{1} \le 7.25,\,\, - 0.35 \le U_{2} \le 0.35$$, $$- 0.35 \le U_{3} \le 0.35,\,\,$$$$- 78.6 \le U_{4} \le 78.6.$$ The ranges of gyro sensor faults are limited as: $$- 5 \le f_{sx} ,\,f_{sy} \le 5,\,$$$$- 0.25 \le f_{sz} \le 0.25$$, while that of actuator faults are limited as: $$- 6.5 \le f_{az} \le 6.5,\,$$ $$- 0.32 \le f_{arol} ,\,f_{apit} \le 0.32,\,$$$$- 0.15 \le f_{ayaw} \le 0.15.$$

### Fault-free case

Desired translational motion is commanded as ${z}_{d}=1\, \text{m}$, ${x}_{d}=1\, \text{m}$, ${y}_{d}=1\, \text{m}$ at $5\,$, $10$, and $20\,{\text{ s}}$, respectively.

**Figure 4:**
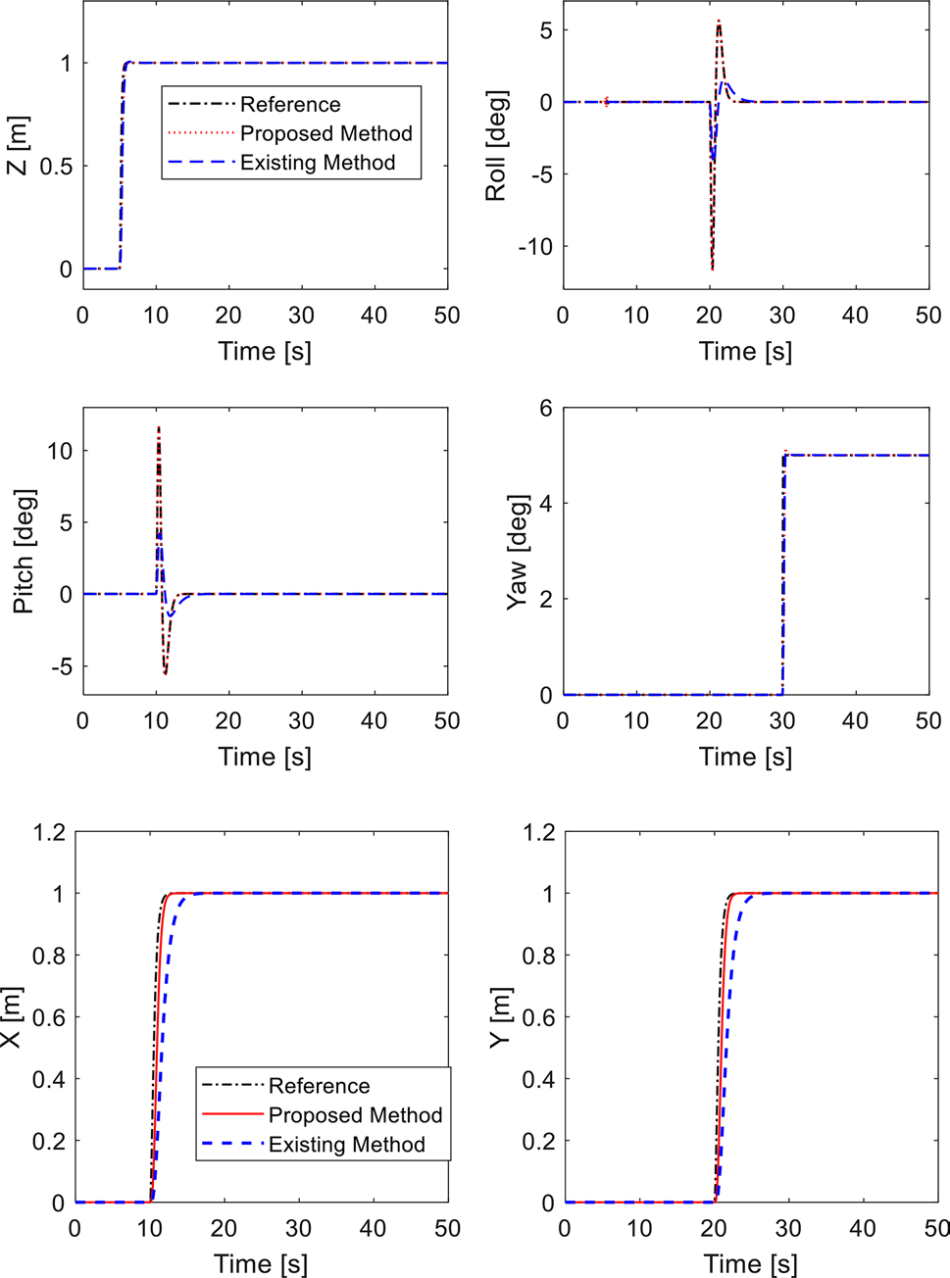
Tracking performance in fault-free case.

**Figure 5:**
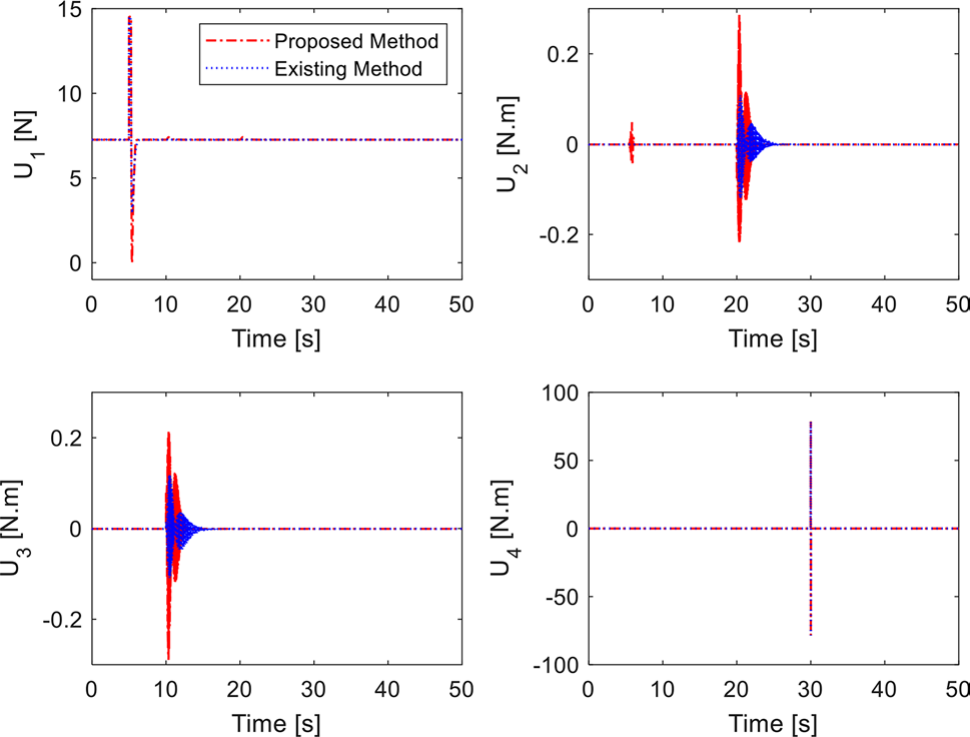
Control inputs in fault-free case.

Desired heading is set as ${\psi }_{d}={5}^{^\circ }$ at $30 \; \text{ s}$. Desired roll and pitch angles are generated through (40). Simulation is performed using our method and^33^ with no fault. The tracking performance is shown in Fig. 4. Both methods show precise tracking during fault-free condition. It is noted that by adding the double reaching law, the performance of roll and pitch from proposed method is much faster than the existing method. Corresponding control inputs are plotted in Fig. 5. Note that the control inputs experience oscillation for a short interval due to step commands of the altitude and attitude angles.

### Sensor fault

**Figure 6:**
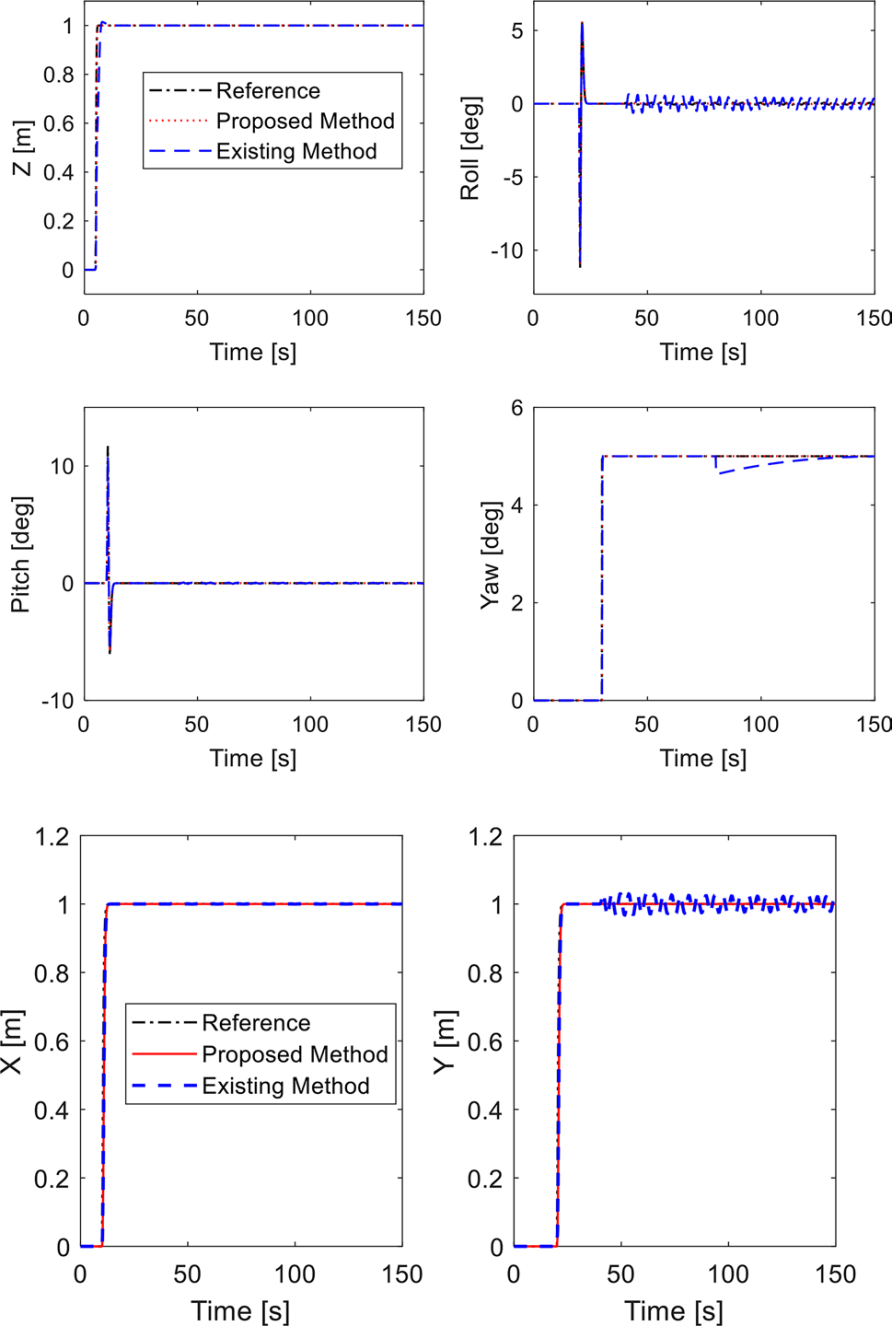
Tracking performance in presence of sensor fault.

**Figure 7:**
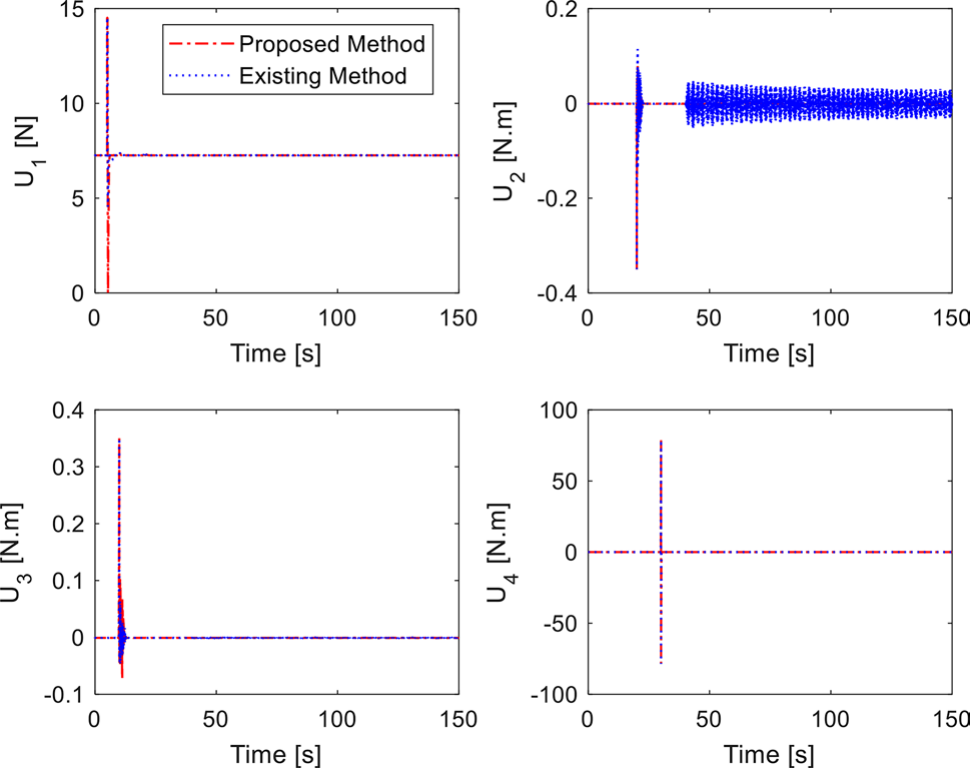
Control inputs in presence of sensor fault.

**Figure 8:**
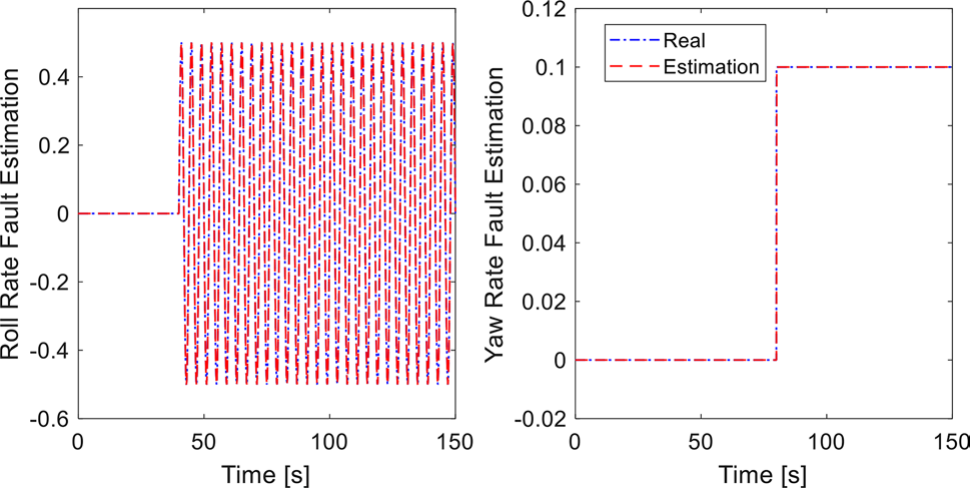
Sensor fault estimation.

In this simulation, the sensor fault signal is injected into gyroscope sensor along the $x$-direction and $z$-direction as:

40 $$\begin{aligned} f_{sx} (t) & = \left\{ \begin{array}{ll} 0 & \quad t \le 40\,\text{s} \hfill \\ 0.5\sin (\pi t/2), & \quad t > 40\, \text{s} \hfill \\ \end{array} \right. \hfill \\ f_{sz} (t) & = \left\{ \begin{array}{ll} 0 & \quad t \le 80\,\text{s} \hfill \\ 0.1, & \quad t > 80\,\text{s} \hfill \\ \end{array} \right. \hfill \\ \end{aligned}$$

while the desired motion commands are the same as before. The tracking performance is shown in Fig. 6. It is evident that the proposed integral sliding mode fault tolerant controller can provide good tracking performance thanks to fault signal compensation from the fault diagnosis observer while the compared method shows oscillation in roll angle, yaw angle and $y$-position when fault occurs at $t=40$ s and $t=80$ s. Control inputs are plotted in Fig. 7. Estimation of the sensor fault signal is depicted in Fig. 8. The proposed fault diagnosis observer can track the actual fault signal quickly. Thus, the fault rejection can be achieved in a timely manner. The root-mean-square-error of two controllers are presented in Table 3 for comparison^45^. It is shown the proposed method is better than compared method under sensor fault.

**Table 3:** Tracking errors in sensor fault.

	Proposed Method	Compared Method
Roll	$0.0009$	$0.0016$
Pitch	$2.043 \times 10^{ - 8}$	$5.869 \times 10^{ - 8}$
Yaw	$6.943 \times 10^{ - 9}$	$4.468 \times 10^{ - 5}$
X	$2.029 \times 10^{ - 6}$	$0.00027$
Y	$0.00054$	$0.0144$
Z	$2.5212 \times 10^{ - 9}$	$1.0853 \times 10^{ - 8}$

### Actuator and sensor fault

**Figure 9:**
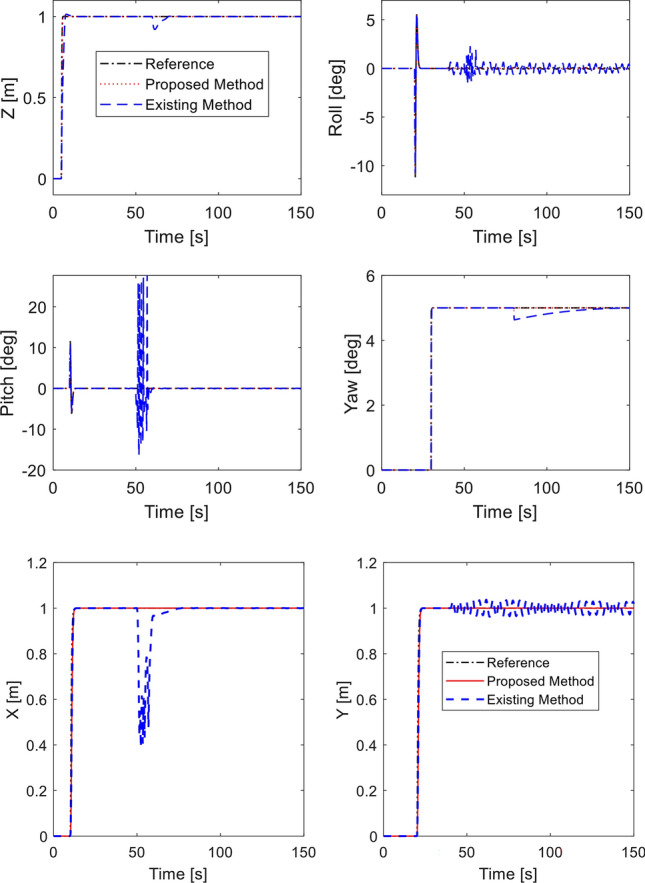
Tracking performance with sensor and actuator fault.

**Figure 10:**
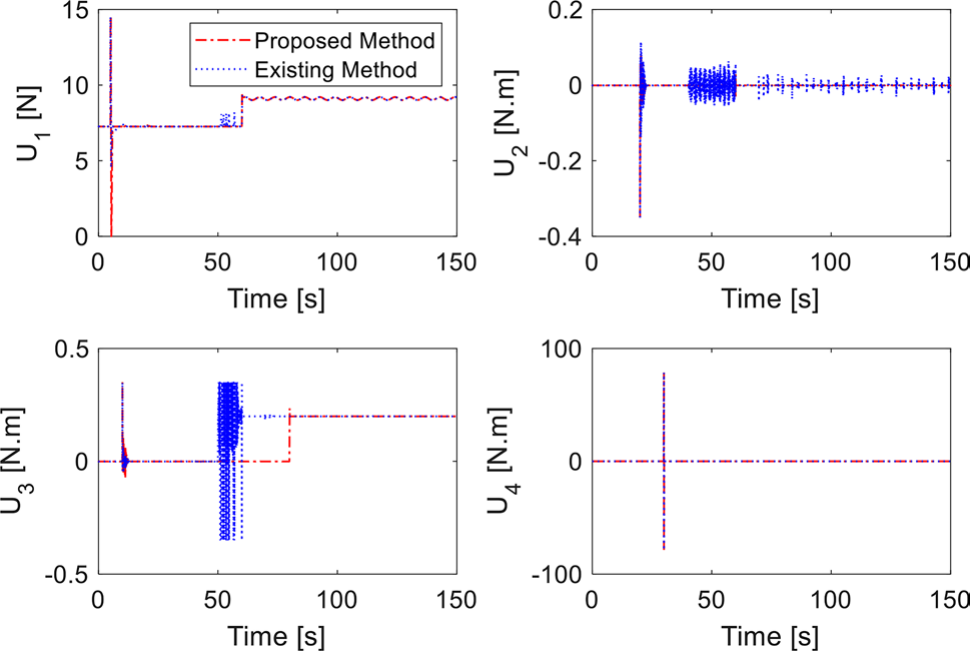
Control inputs in presence of sensor and actuator fault.

**Figure 11:**
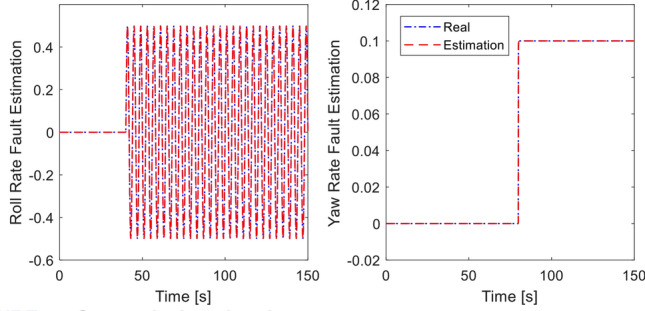
Sensor fault estimation.

**Figure 12:**
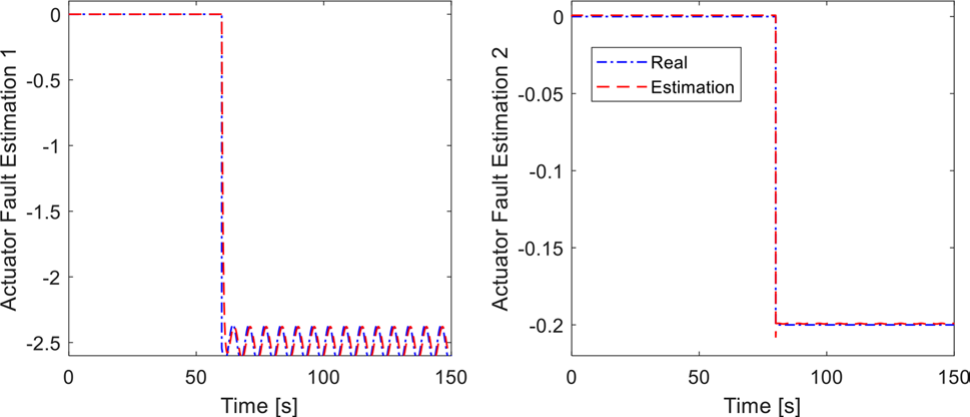
Actuator fault estimation.

In the last simulation, actuator fault signal is introduced in addition to the earlier sensor fault signal. We assume that loss of control effectiveness (LoCE) in pitch moment and *z*- moment occurs at $t = 60\,{\text{ s}}$ and $t = 80\,{\text{ s}}$ as $f_{apit} = 0.2$ and $f_{az} = 2.5 + 0.135\sin (\pi t/4)$. The tracking performance is shown in Fig. 9. At $t = 50\,{\text{ s}}$ where the *z*- moment is lost, the proposed controller still provides fast tracking performance thanks to the fault signal compensation, double reaching law. However, the method of^33^ shows large deviation from the desired position due to ocssillation of roll and pitch response. Without fault signal compensation, the closed loop dynamics spends long recovery time before settling, as seen from the $x$-motion plot. Control inputs are plotted in Fig. 10. It should be noted that the compared method experiences large and prolonged oscillation due to multiple faults (sensor and actuator faults). Estimation of sensor and actuator faults are shown in Figs. 11 and 12. The observer correctly estimate the actuator fault values and the sensor fault of sinusoidal signal generated from (40). The root-mean-square-error of both methods are presented in Table 4. It is shown the proposed method is better than compared method under sensor fault and actuator fault.

**Table 4:** Tracking errors in actuator and sensor faults.

	Proposed method	Compared method
Roll	$0.00091$	$0.00166$
Pitch	$4.155 \times 10^{ - 9}$	$6.249 \times 10^{ - 7}$
Yaw	$1.292 \times 10^{ - 8}$	$4.486 \times 10^{ - 5}$
X	$3.839 \times 10^{ - 6}$	$0.0005$
Y	$0.0007$	$0.0193$
Z	$9.6216 \times 10^{ - 9}$	3.325 × 10^−6^

## Conclusions

In this paper, an active fault tolerant control approach is proposed to resolve the effects of both actuator and sensor faults in the UAVs. We introduce the fault diagnosis observer that can estimate the sensor and actuator fault signals. Considering the control input saturation, we design an integral sliding mode fault tolerant controller that uses the estimated fault signals to compensate for the faults appropriately. Radial basis function neural network is applied in the controller to overcome the model uncertainties. The Lyapunov theorem is applied to prove the stability of the observer and controller. The efficacy of this approach is demonstrated through simulations. The results show the proposed method outperforms the baseline controller in tracking performance. This improvement is attributed to the compensation for fault effects through the fault estimation and the sliding mode fault controller with anti-saturation algorithm. Our future work will be to realize the proposed method in the actual UAV systems.

## Appendix

41 $$K_{1} = K_{2} = 10^{6} \times \left[ {\begin{array}{*{20}c} { - 0.0148} & 0 & 0 & 0 & {0.0009} & 0 & 0 & 0 \\ 0 & { - 3.779} & 0 & 0 & 0 & {0.2195} & 0 & 0 \\ 0 & 0 & { - 3.779} & 0 & 0 & 0 & {0.2195} & 0 \\ 0 & 0 & 0 & { - 1.8005} & 0 & 0 & 0 & {0.1045} \\ \end{array} } \right]$$

42 $$\, L_{0} = {10}^{4} \, \times \left[ {\begin{array}{*{20}c} {0.0223} & 0 & 0 & 0 & {0.0001} & 0 & 0 & 0 \\ 0 & {0.0224} & 0 & 0 & 0 & {0.0001} & 0 & 0 \\ 0 & 0 & {0.0224} & 0 & 0 & 0 & {0.0001} & 0 \\ 0 & 0 & 0 & {{0}{\text{.0224}}} & 0 & 0 & 0 & {00.0001} \\ {{ 4}{\text{.2140}}} & 0 & 0 & 0 & {{0}{\text{.0181}}} & 0 & 0 & 0 \\ 0 & {4.1982} & 0 & 0 & 0 & {{0}{\text{.0173}}} & 0 & 0 \\ 0 & 0 & {4.1982} & 0 & 0 & 0 & {{0}{\text{.0173}}} & 0 \\ 0 & 0 & 0 & {4.1994} & 0 & 0 & 0 & {{0}{\text{.0174}}} \\ { - 0.0225} & 0 & 0 & 0 & {0.0001} & 0 & 0 & 0 \\ 0 & { - 0.022} & 0 & 0 & 0 & {0.0001} & 0 & 0 \\ 0 & 0 & { - 0.0225} & 0 & 0 & 0 & {0.0001} & 0 \\ 0 & 0 & 0 & { - 0.0225} & 0 & 0 & 0 & {0.0001} \\ \end{array} } \right]$$

43 $$P = 10^{7} \times \left[ {\begin{array}{*{20}c} {{0}{\text{.5653}}} & 0 & 0 & 0 & { - 0.0015} & 0 & 0 & 0 & {0.1245} & 0 & 0 & 0 \\ 0 & {0.566} & 0 & 0 & 0 & { - 0.0015} & 0 & 0 & 0 & {0.1224} & 0 & 0 \\ 0 & 0 & {0.566} & 0 & 0 & 0 & { - 0.0015} & 0 & 0 & 0 & {0.1224} & 0 \\ 0 & 0 & 0 & {0.5661} & 0 & 0 & 0 & { - 0.0015} & 0 & 0 & 0 & {0.1223} \\ { - 0.0015} & 0 & 0 & 0 & {0.0001} & 0 & 0 & 0 & {0.0128} & 0 & 0 & 0 \\ 0 & { - 0.0015} & 0 & 0 & 0 & {0.001} & 0 & 0 & 0 & {0.0132} & 0 & 0 \\ 0 & 0 & { - 0.0015} & 0 & 0 & 0 & {0.0001} & 0 & 0 & 0 & {0.0132} & 0 \\ 0 & 0 & 0 & { - 0.0015} & 0 & 0 & 0 & {0.0001} & 0 & 0 & 0 & {0.0132} \\ {0.1245} & 0 & 0 & 0 & {0.0128} & 0 & 0 & 0 & {2.5248} & 0 & 0 & 0 \\ 0 & {0.1224} & 0 & 0 & 0 & {0.0132} & 0 & 0 & 0 & {2.5800} & 0 & 0 \\ 0 & 0 & {0.1224} & 0 & 0 & 0 & {0.0132} & 0 & 0 & 0 & {2.58} & 0 \\ 0 & 0 & 0 & {0.1223} & 0 & 0 & 0 & {0.0132} & 0 & 0 & 0 & {2.5802} \\ \end{array} } \right]$$
